# The activation of M3 mAChR in airway epithelial cells promotes IL-8 and TGF-β1 secretion and airway smooth muscle cell migration

**DOI:** 10.1186/s12931-016-0344-5

**Published:** 2016-03-08

**Authors:** Juan-Juan Lu, Guang-Ni Xu, Ping Yu, Yun Song, Xiao-Lin Wang, Liang Zhu, Hong-Zhuan Chen, Yong-Yao Cui

**Affiliations:** Department of Pharmacology, Shanghai Jiao Tong University School of Medicine, 280 South Chongqing Road, Shanghai, 200025 China; Department of Anatomy, Histology and Embryology, Shanghai Jiao Tong University School of Medicine, Shanghai, China

**Keywords:** ASM cell migration, Chemokines, Human epithelial cells, Non-neuronal cholinergic system, Signaling pathways

## Abstract

**Background:**

Muscarinic acetylcholine receptors (mAChRs) have been identified in airway epithelium, and epithelium-derived chemokines can initiate the migration of airway smooth muscle (ASM) cells. However, the mAChRs that are expressed in airway epithelium and the mechanism underlying the regulation of ASM cell migration are not clear. The aim of this study was to test whether the effects of the epithelium-derived chemokines on ASM cell migration could be modulated by mAChRs.

**Method:**

Human epithelial cells (A549 cells) were stimulated with cigarette smoke extract (CSE) or the mAChRs agonist carbachol. IL-8 and TGF-β1 production were measured by ELISA, and human ASM cell migration was measured using the transwell migration assay and scratch assay. The mRNA levels of the mAChRs subtypes and the acetylcholine concentrations were measured using RT-PCR and LC–MS/MS, respectively.

**Results:**

ASM cell migration toward CSE-stimulated A549 cells was markedly reduced by Ac-RRWWCR-NH2 (IL-8 inhibitor) and SB431542 (TGF-β1 inhibitor). CSE-induced ASM cell migration was also suppressed by the mAChRs antagonist tiotropium. Interestingly, carbachol-stimulated A549 cells also induced ASM cell migration; this migration event was suppressed by tiotropium, Ac-RRWWCR-NH2 and SB431542. In addition, the effects of CSE on ASM cell migration were significantly and cooperatively enhanced by carbachol compared to CSE alone. Carbachol-induced ASM cell migration was reduced by selective inhibitors of PI3K/Akt (LY294002) and p38 (SB203580), suggesting that it occurred through p38 and Akt phosphorylation, which was inhibited by the M3 mAChR antagonist 4-DAMP.

**Conclusions:**

These findings indicate that M3 mAChR may be important therapeutic target for obstructive airway diseases, as it regulates the effects of the epithelial-derived chemokines on ASM cell migration, which results in lung remodeling.

## Background

Airway remodeling is one of the major features of many obstructive airway diseases, and has been observed both in asthma and in chronic obstructive pulmonary disease (COPD) [1, 2]. The adverse consequences of airway remodeling could include a decrease in lung function and reduced responsiveness to bronchodilator therapy. Different mechanisms were proposed to explain smooth muscle remodeling, including proliferation (hyperplasia), an increase in myocyte mass and size and an increase in their components as a response to injury or inflammation [3, 4]. However, many studies have suggested that the migration of ASM cells toward the lumen of the airway might contribute to ASM remodeling [5–10] and plays an important role in the pathophysiology of airway remodeling [8, 9, 11–13].

It has been reported that cigarette smoke or cigarette smoke condensate induced tracheal and lung epithelial cell damage and shedding. Lung epithelial cells are not only the simple structural cells of the lung but also serve as a barrier for inhaled particles and noxious gases (such as cigarette smoke). These cells are also actively involved in inflammatory and repair processes through the release of cytokines and growth factors that maintain normal airway homeostasis and play an important role in the prevention of pulmonary diseases. Airway remodeling may represent the effect of cigarette smoke on the epithelium and the attempts by the airway epithelium to protect itself and repair the injury caused by cigarette smoke. The human alveolar epithelial cell line A549 (A549) is widely acknowledged to be a relevant model cell line for biological evaluations of this mechanism [14, 15].

Airway smooth muscle (ASM) cells are associated with asthma severity and COPD and have the capacity to migrate. Many chemokines, such as growth factors and cytokines, have been implicated in the initiation and the perpetuation of ASM cell migration [11, 16]. In fact, a strong relationship between chronic injury or defective repair of the airway epithelium and airway remodeling has been reported in the literature [17]. Not surprisingly, the epithelium is a major source of the cytokines and growth factors that act as mitogens for ASM cells, such as IL-8 and TGF-β1. More important to the study of epithelial-ASM cell interactions, the receptors for epithelial-derived growth factors and cytokines, such as TGF-β1 and IL-8, were identified on the ASM cell surface [18, 19]. Increased expression of the TGF-β1 and IL-8 mRNAs and proteins has been observed in the bronchiolar epithelium of smokers with COPD compared to smokers without COPD [20–22]. The expression of TGF-β1 at the injury site may have a central role in tissue homeostasis and repair. IL-8 is a potent neutrophil chemoattractant [23] and a potential contributor to airway remodeling, as it induces airway smooth muscle proliferation and migration [16].

Airway epithelial damage exposes the sensory nerve endings to the airway lumen and promotes reflex mechanisms, leading to enhanced vagal release of acetylcholine [24]. Recent findings suggest that the acetylcholine production in the airways is not restricted to the parasympathetic nervous system, but can also be released from non-neuronal cells and tissues, such as the airway epithelium, to regulate aspects of inflammation and remodeling through its action on mAChRs [25–29]. Dysfunction of the non-neuronal cholinergic system appears to be involved in the pathophysiology of asthma and COPD. Although a number of molecules that are involved in acetylcholine-mediated airway inflammation and remodeling have been identified, the net effect of mAChRs in regulating epithelial-derived chemokine-induced ASM cell migration has not been explored in great detail.

The aim of the present study was to characterize the potential role of the non-neuronal components of the cholinergic system on ASM cell migration toward bronchial epithelial cells stimulated with CSE. For this reason, we first evaluated whether the IL-8 and TGF-β1 from the CSE-stimulated epithelial cells might be able to initiate ASM cell migration using the transwell assay and the co-culture model of human ASM cells and A549 cells. Then, we tested whether the ability of CSE to induce the ASM cell migration might be regulated by mAChRs and reproduced by an exogenous mAChRs agonist, and determined their mechanism.

## Methods

### Cell culture

The A549 cells obtained from the American Type Culture Collection (Manassas, VA, USA) and the normal primary human airway smooth muscle cells (passage 3-8, from a single healthy donor) obtained from Pricell Inc. (Shanghai, China) were cultured in Ham’s F-12 medium (Gibco, USA) and Dulbecco’s Modified Eagle’s Medium (DMEM), respectively, containing 10 % fetal bovine serum, L-glutamine (2 mM), penicillin (100 U/ml) and streptomycin sulfate (100 U/ml, Gibco, USA) in a humidified incubator at 37 °C with 5 % CO_2_.

### Cigarette Smoke Extract (CSE)

The CSE was prepared as previously described, with minor modifications [30]. The CSE was prepared by combusting 1 cigarette (double happiness, the amount of tar was 12 mg), using a pump and passing the smoke through 10 ml of FBS-free culture medium at a rate of 5 min/cigarette. The resulting solution was adjusted to pH 7.4 with 1 mol/L of concentrated NaOH and filtered through a 0.22-μm filter. The obtained solution was referred to as 100 % strength and diluted to the desired concentrations with culture medium.

### Measurement of the IL-8 and TGF-β1 produced by the A549 cells

After serum deprivation for 24 h before each experiment, the cells were pre-treated with tiotropium (Sigma–Aldrich, USA) for 30 min before 3 % CSE stimulation, or treated with different concentrations of carbachol alone. After 72 h, the concentrations of IL-8 and TGF-β1 in the culture supernatants were immediately measured using an ELISA kit (R&D Systems, Minneapolis, MN) according to the manufacturer’s protocol.

### Cell migration assay

We jointly applied transwell migration chamber assay and scratch assay to examine the ASM cell migration induced by epithelial derived supernatants. For transwell migration chamber assay, the A549 cells were cultured in the lower wells (omitted in the negative control) at densities of 100,000 cells/cm^2^ and grown to 80 % confluence, followed by serum-deprivation for 24 h before each experiment. Subsequently, the cells were treated with carbachol or 3 % CSE for 72 h. Tiotropium (Tio, 0.1, 1, 10 μM) and 4-DAMP (10 μM, selective M3 mAChR antagonist) were added to the cells 30 min before CSE stimulation and were present throughout the experiment. Additionally, where appropriate, the A549 cells were pre-incubated with either the IL-8 inhibitor Ac-RRWWCR-NH2 (AnaSpec, Inc., Fremont, Calif) or the TGF-β1 inhibitor SB431542 (Sigma–Aldrich, USA) for 30 min. The ASM cells (30,000 cells/cm^2^) were added to the upper chambers. The vehicle was DMSO and the final concentration of DMSO was 0.1 % in all wells, including the controls. After incubating the cells for 6 h at 37 °C, the upper chamber was removed from the 24-well plates and the residual cells on the upper side of the transwell filters were wiped with cotton swab. The transwell filters were rinsed with PBS and fixed in 4 % formaldehyde in PBS for 15 min. The cells that had migrated to the lower surface were stained with 0.1 % crystal violet for 25 min, visualized and photographed under an inverted fluorescence microscope (Zeiss, Germany). The migration rate was analyzed using the Image Pro Plus 6.0 software.

For scratch assay, the confluent A549 cells were stimulated with 3 % CSE for 72 h with the pretreatment of Ac-RRWWCR-NH2 and SB431542. Then, the supernatants were collected as stimulants for ASM cell migration. ASM cells were cultured in DMEM until 100 % confluence and then wounded with a sterile 20 μL pipette tip. Subsequently, the cells were washed with PBS and treated with the epithelial derived supernatants. The wounds were photographed at baseline and 24 h later under an inverted fluorescence microscope (AMG, America). The wound healing width was analyzed using the Image Pro Plus 6.0 software.

### Determination of the acetylcholine levels by LC-MS/MS

The acetylcholine levels in the supernatants from the A549 cells were determined by LC–MS/MS, as previously described [31].

### Real-time quantitative PCR of the mAChRs

The A549 cells were stimulated with 3 % CSE for 24, 48 or 72 h. The expression of the mAChRs was detected by Real-time quantitative PCR (RT-PCR), as previously described [31].

### Western blot analysis

Equal amounts of protein were loaded onto 10 % SDS-PAGE gels and transferred to polyvinylidene fluoride membranes. To avoid non-specific binding, the membranes were blocked with 5 % non-fat milk in TBS-T (Tris-HCl 50 mM, NaCl 150 mM, and Tween-20 0.1 %) for 1 h at room temperature. The membranes were then incubated with primary antibodies against phosphorylated-p38 (#4511; Cell Signaling Technology, USA) or phosphorylated-Akt (#4060; Cell Signaling Technology, USA) overnight at 4 °C. After washing three times with TBS-T for 10 min, the membranes were incubated with an HRP-conjugated secondary antibody (1:5000) for 1 h at room temperature, followed by three additional washes with TBS-T. The bands were subsequently visualized on film using enhanced chemiluminescent reagents. All bands were normalized against the total p38 (for phospho-p38) and total Akt (for phospho-Akt) levels, as appropriate. In the experiments designed to establish whether the p38 MAPK or PI3K/Akt pathways were involved in the ASM cell migration induced by the mAChRs agonist carbachol, a p38 inhibitor (SB203580: 10 μM) or a PI3K/Akt inhibitor (LY294002: 10 μM) was added to the culture 1 h prior to stimulation.

### Statistical analysis

The data are presented as the means ± SEM. The statistical significance of the differences between groups was assessed with one-way analysis of variance (ANOVA), followed by a Dunnett’s test for selected pairs, as appropriate. *p*-values <0.05 were considered significant. All statistical analyses were performed using Prism version 5.0 (GraphPad Software, San Diego, USA).

## Results

### The role of IL-8 and TGF-β1 from the CSE-stimulated epithelial cells in initiating ASM cell migration

**Fig. 1:**
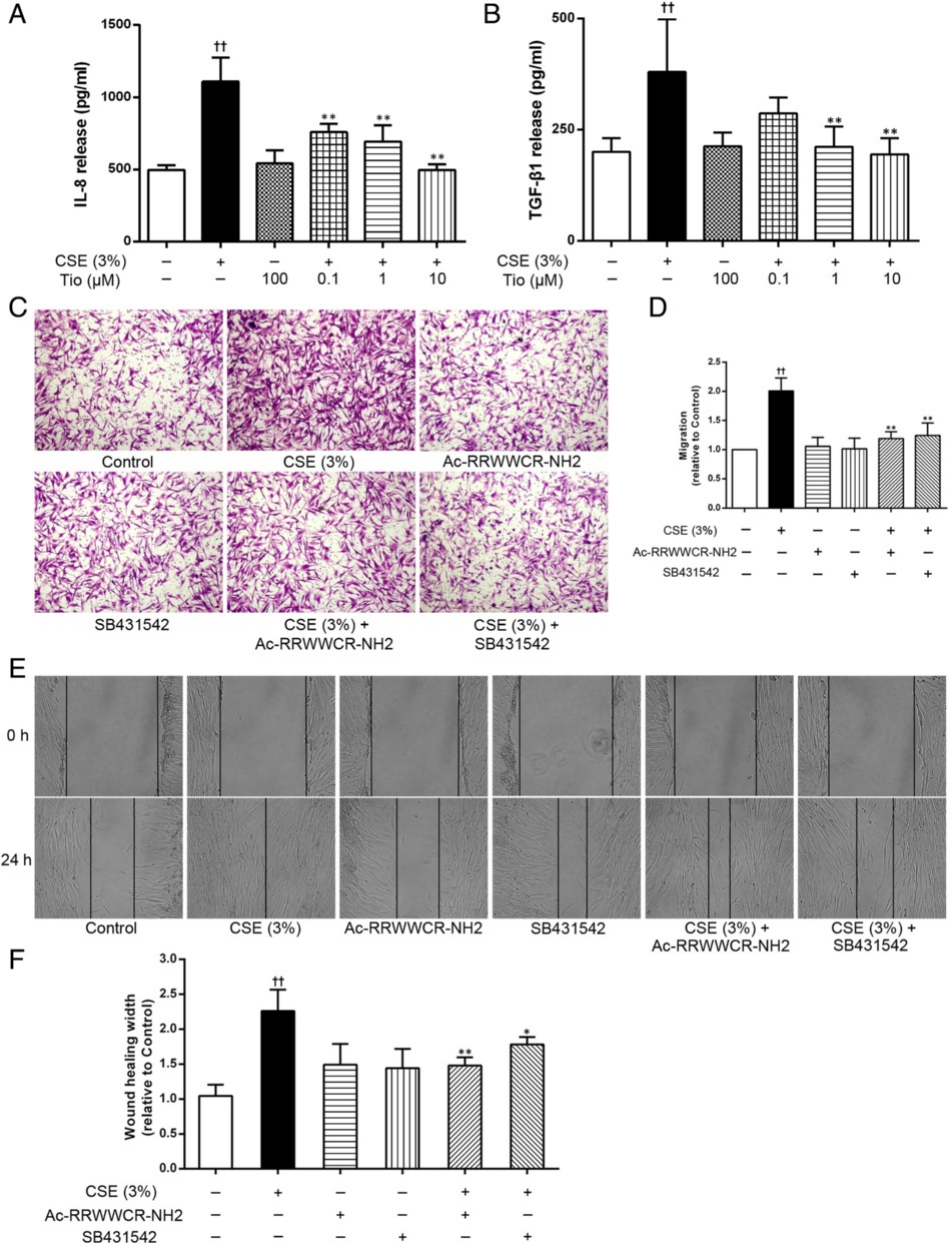
The role of IL-8 and TGF-β1 from CSE-stimulated epithelial cells in initiating ASM cell migration. Tiotropium (Tio, 0.1, 1, 10 μM) was added to the A549 cells 30 min before stimulation with 3 % CSE. After 72 h of stimulation, the supernatants were collected and the IL-8 (**a**) and TGF-β1 (**b**) concentrations were determined using an ELISA. ASM cell migration initiated by the A549 cells stimulated with 3 % CSE were reduced by the IL-8 inhibitor Ac-RRWWCR-NH2 and the TGF-β1 inhibitor SB431542. Cell migration was detected by using transwell chamber assay (**c**, ×100) and scratch assay (**e**, ×100). A quantitative analysis of migration used Image Pro Plus 6.0 (**d** and **f**). The values are expressed as the means ± SEM, *n* = 3-5. ^††^ *p* < 0.01 vs the control group, ^*^ *p* < 0.05 and ^**^ *p* < 0.01 vs the CSE group

The A549 cells that had been activated with 3 % CSE exhibited increased IL-8 and TGF-β1 levels in the supernatants, resulting in ASM cell migration toward the A549 cells. The IL-8 or TGF-β1 levels in supernatants were increased by approximately 2.23-fold and 1.89-fold, respectively, in response to CSE stimulation (*p* <0.01), that effect was markedly inhibited by tiotropium in a concentration-dependent manner (Fig. 1a and b). We also used the transwell assay and the scratch assay to further assess whether CSE-induced ASM cell migration was initiated by IL-8 or TGF-β1. The IL-8 inhibitor Ac-RRWWCR-NH2 or the TGF-β1 inhibitor SB431542 was added to the lower chamber of the transwell system (A549 epithelial cells). As expected, the results from transwell migration assay suggested that Ac-RRWWCR-NH2 and SB431542 decreased ASM cell migration ratio by 81.06 and 75.81 % (*p* <0.01) compared to the non-treated cells, indicating that the compounds inhibited ASM cell migration (Fig. 1c and d). As results of transwell assay, scratch assay also demonstrated that supernatants from CSE-stimulated epithelial cells induced ASM cell migration, this phenomenon was significantly inhibited by both Ac-RRWWCR-NH2 and SB431542 (Fig. 1e and f), suggesting that the IL-8 and TGF-β1 from the CSE-stimulated A549 cells have an important role in driving ASM cell migration.

### Blockade of the epithelial cell-derived chemokines by an mAChRs antagonist reduces ASM cell migration

**Fig. 2:**
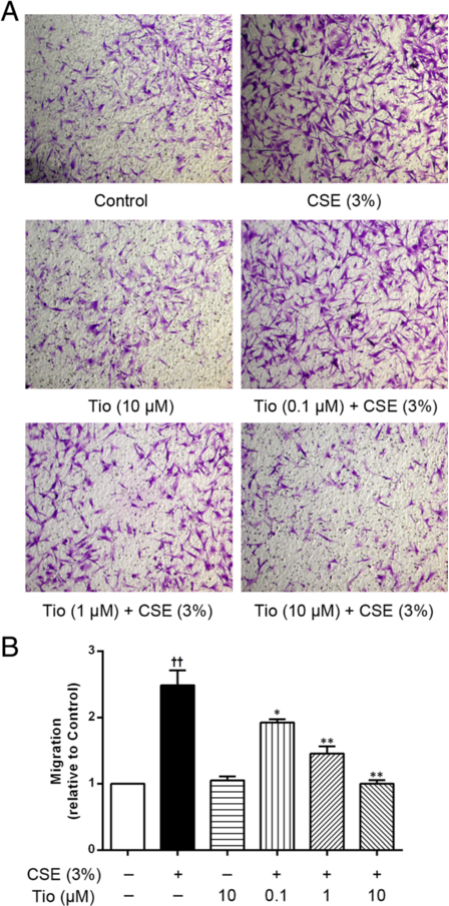
Effects of tiotropium on ASM cell migration initiated by the A549 cells stimulated with CSE. The A549 cells were stimulated with 3 % CSE for 72 h. Tio (0.1, 1, 10 μM) was added to the cells 30 min before 3 % CSE stimulation and was present throughout the experiment. ASM cell migration initiated by the stimulated A549 cells was detected after 6 h of co-culture in the transwell system, the migrated ASM cells were stained with crystal violet (**a**, ×100). A quantitative analysis of migration used Image Pro Plus 6.0 (**b**). The values are expressed as the means ± SEM, *n* = 3. ^††^ *p* < 0.01 vs the control group. ^*^ *p* < 0.05 and ^**^ *p* < 0.01 vs the CSE group

As airway epithelial cells exhibit all components of the non-neuronal cholinergic system, we investigate the involvement of this system in the control of ASM cell migration in response to the epithelial cell-derived chemokines. The A549 cells were pre-incubated with the mAChRs antagonist tiotropium which was added 30 min before CSE stimulation. As shown in Fig. 2, the CSE-stimulated A549 cell-induced ASM cell migration was significantly reduced by tiotropium in a concentration-dependent manner. The ability of tiotropium (0.1, 1, 10 μM) to regulate the ASM cell migration was 37.53, 69.32 and 89.96 % lower than the cells treated with 3 % CSE alone, respectively, suggesting that the endogenous acetylcholine exerts its activity by activating the mAChRs on the epithelial cells.

### Involvement of the non-neuronal cholinergic system in epithelial-derived chemokine-mediated ASM cell migration

**Fig. 3:**
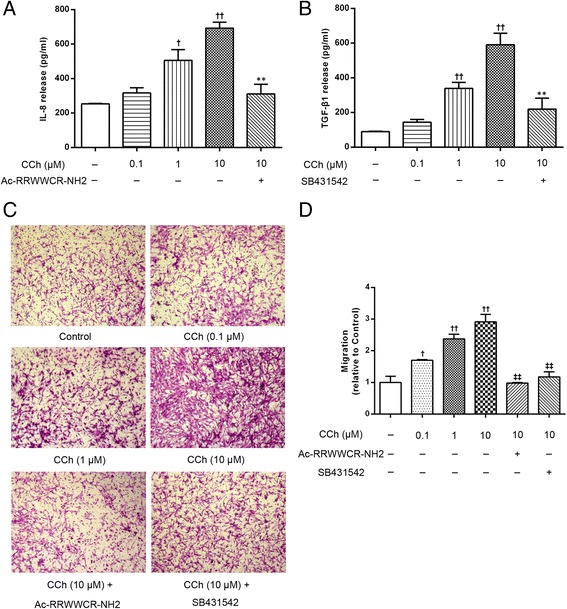
The carbachol-stimulated A549 cells induced IL-8 and TGF-β1 release and ASM cell migration. The A549 cells were stimulated with carbachol (CCh, 0.1, 1, 10 μM) in the presence or absence of the IL-8 inhibitor Ac-RRWWCR-NH2 and the TGF-β1 inhibitor SB431542 for 72 h. Then, the supernatants were collected and the IL-8 (**a**) and TGF-β1 (**b**) concentrations were determined using an ELISA. ASM cell migration initiated by the stimulated A549 cells was detected after 6 h of co-culture in the transwell system, the migrated ASM cells were stained with crystal violet (**c**, ×100). A quantitative analysis of migration used Image Pro Plus 6.0 (**d**). The values are expressed as the means ± SEM, *n* = 5–8. ^†^ *p* < 0.05 and ^††^ *p* < 0.01 vs the control group, ^**^ *p* < 0.01 vs the CCh (10 μM) group, ^‡‡^ *p* < 0.01 vs the CCh (10 μM) group

**Fig. 4:**
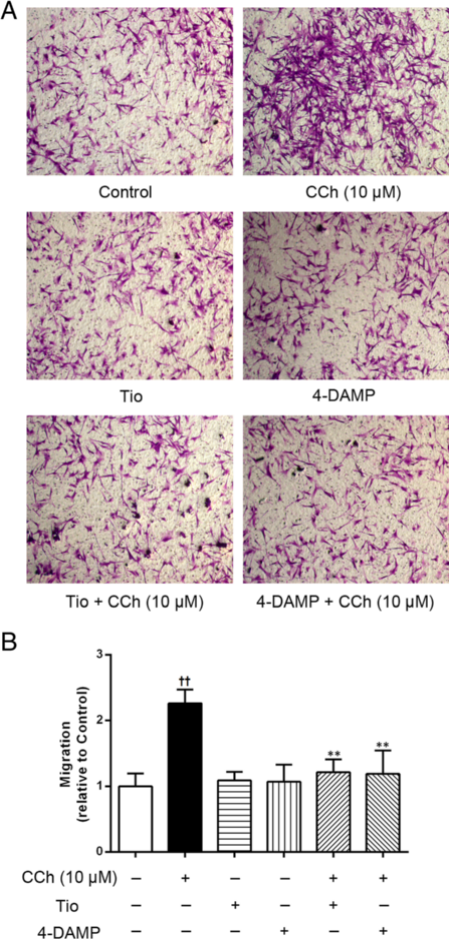
Effects of the mAChR antagonists on carbachol-induced ASM cell migration. The A549 cells were stimulated with CCh (10 μM) for 72 h. Tio (10 μM) or 4-DAMP (10 μM) was added to the cells 30 min before CCh stimulation and was present throughout the experiment. ASM cell migration initiated by the stimulated A549 cells was detected after 6 h of co-culture in the transwell system, the migrated ASM cells were stained with crystal violet (**a**, ×100). A quantitative analysis of migration used Image Pro Plus 6.0 (**b**). The values are expressed as the means ± SEM, *n* = 4. ^††^ *p* < 0.01 vs the control group. ^**^ *p* < 0.01 vs the CCh group

**Fig. 5:**
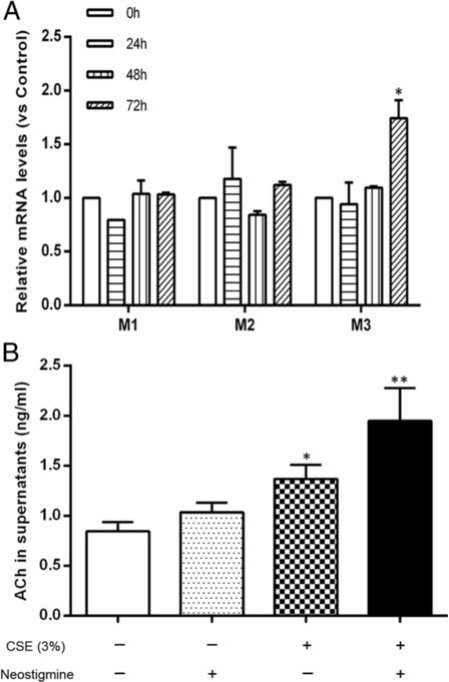
Effects of the CSE on acetylcholine release and the mRNA levels of M1, M2, M3. **a** Neostigmine (10 μM) was added to the A549 cells 1 h before stimulation with 3 % CSE. The acetylcholine (ACh) levels were determined after 72 h co-culture using an LC-MS/MS assay. **b** The A549 cells were incubated with 3 % CSE for 24, 48, 72 h, and the mRNA levels were determined using a quantitative RT-PCR and standardized to that of the GAPDH gene. The values are expressed as the means ± SEM. *n* = 3-5. ^*^ *p* < 0.05 and ^**^ *p* < 0.01 vs the control group

**Fig. 6:**
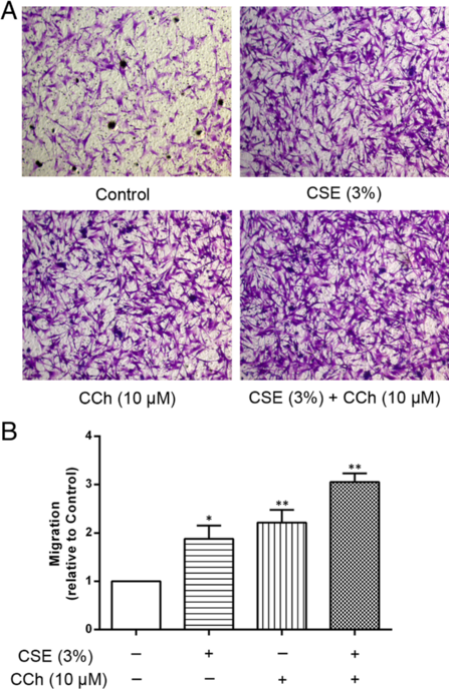
Enhancement of ASM cell migration by co-stimulating the A549 cells with CSE and carbachol. The A549 cells were incubated with 3 % CSE and CCh (10 μM) for 72 h. ASM cell migration initiated by the stimulated A549 cells was detected after 6 h of co-culture in the transwell system, the migrated ASM cells were stained with crystal violet (**a**, ×100). A quantitative analysis of migration used Image Pro Plus 6.0 (**b**). The values are expressed as the means ± SEM, *n* = 4. ^*^ *p* < 0.05 and ^**^ *p* < 0.01 vs the control group

If the non-neuronal cholinergic system in epithelial cells is indeed involved in the epithelial cell-derived chemokine-mediated ASM cell migration, we hypothesized that the application of exogenous mAChRs agonists would reproduce the effect of endogenous acetylcholine. To verify this hypothesis, the A549 cells were first stimulated with the acetylcholine analogue carbachol. As shown in Fig. 3, stimulation of the A549 cells with the mAChRs agonist carbachol increased IL-8 (Fig. 3a) and TGF-β1 (Fig. 3b) levels and induced concentration-dependent ASM cell migration, which could be inhibited by the IL-8 inhibitor Ac-RRWWCR-NH2 and the TGF-β1 inhibitor SB431542, respectively (Fig. 3c and d). Moreover, carbachol-induced ASM cell migration was significantly reduced by tiotropium (10 μM) (*p* <0.01) and 4-DAMP (10 μM) (*p* <0.01) (Fig. 4). These results strongly support our hypothesis that endogenous acetylcholine may stimulate the M3 mAChR in A549 cells to promote ASM cell migration. We also found that the stimulation of A549 cells with CSE increased the expression of the M3 mAChR mRNA (Fig. 5a), as well as acetylcholine production. Interestingly, we also found that CSE-induced acetylcholine release was increased by 42.34 % in the presence of a cholinesterase inhibitor neostigmine, which did not affect acetylcholine release when using alone (Fig. 5b). Because the inducing effect of endogenous acetylcholine could be reproduced by an exogenous mAChRs agonist, we further hypothesized that the stimulatory effect of CSE on ASM cell migration could be enhanced by carbachol. Thus, co-incubation experiments were performed using carbachol (10 μM) and 3 % CSE. CSE-induced ASM cell migration was significantly and cooperatively enhanced by carbachol compared to the cells stimulated with CSE alone. And a synergistic effect of CSE plus carbachol on ASM cell migration was observed in comparison with CSE or carbachol alone (Fig. 6). The enhancing effect of carbachol prompts us to propose that an autocrine/paracrine loop for acetylcholine and the activation of M3 mAChR regulates ASM cell migration.

### The role of the p38 and PI3K/Akt signaling pathways in mAChR-mediated ASM cell migration

**Fig. 7:**
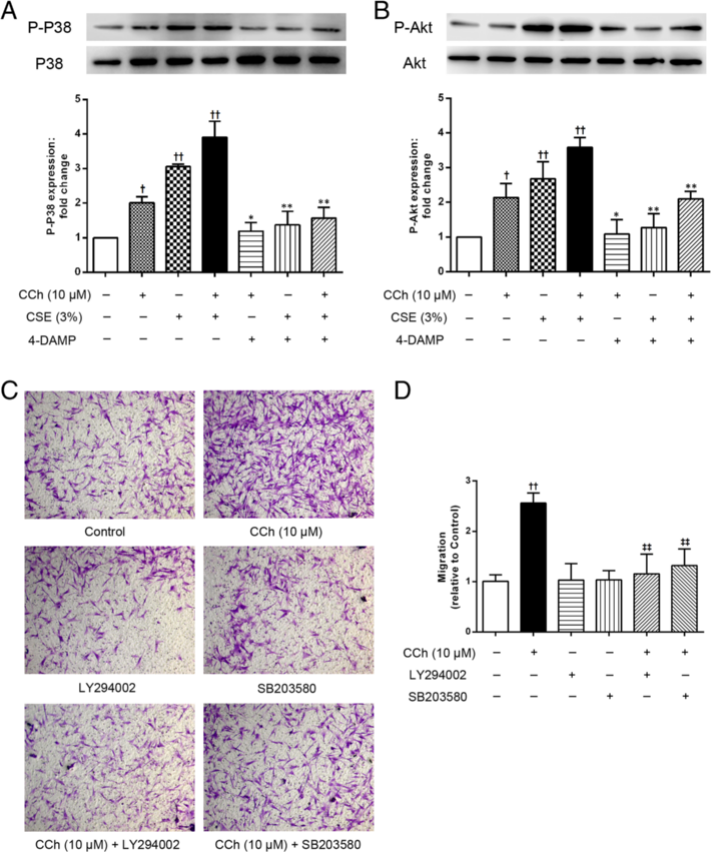
The involvement of the p38 MAPK and PI3K/Akt pathways in ASM cell migration. **a** and **b** The A549 cells were co-incubated with CCh (10 μM) or 3 % CSE for 72 h after pretreatment with 4-DAMP (10 μM). Then the cell lysates were assayed for the levels of total and phosphorylated p38 and Akt; the total p38 and Akt levels were used as a loading control. The A549 cells were incubated with CCh (10 μM) for 72 h after pretreatment with LY294002 (10 μM) or SB203580 (10 μM). ASM cell migration initiated by the stimulated A549 cells was detected after 6 h of co-culture in the transwell system, the migrated ASM cells were stained with crystal violet (**c**, ×100). A quantitative analysis of migration used Image Pro Plus 6.0 (**d**). The values are expressed as the means ± SEM. *n* = 4–6. ^†^ *p* < 0.05 and ^††^ *p* < 0.01 vs the control group, ^*^ *p* < 0.05 and ^**^ *p* < 0.01 vs the corresponding group without 4-DAMP. ^‡‡^ *p* < 0.01 vs the CCh group

To better understand how mAChRs mediated ASM cell migration, the downstream signaling pathways that were activated by the mAChR agonist or CSE were investigated. We first assessed the relationship between mAChR activation and the p38 and PI3K/Akt pathways on ASM cell migration using an mAChR agonist and antagonist. As shown in Fig. 7a and b, the phosphorylation of both p38 and Akt was increased by the mAChR agonist carbachol (10 μM, *p* <0.05) and CSE-induced phosphorylation of p38 and PI3K/Akt were enhanced by carbachol (*p* <0.01). In addition, the carbachol- or carbachol plus CSE-induced phosphorylation of p38 and Akt was significantly inhibited by 4-DAMP, suggesting that there is a relationship between the M3 mAChR and the p38 and PI3K/Akt pathways in ASM cell migration. Specific inhibitors of p38 and PI3K/Akt were used to further confirm the contribution of p38 and PI3K/Akt activation to ASM cell migration. As shown in Fig. 7c and d, SB203580 (10 μM, *p* <0.01) and LY294002 (10 μM, *p* <0.01) caused marked reductions in carbachol-induced ASM cell migration, suggesting that the p38 and PI3K/Akt signaling pathways are required for ASM cell migration. These findings indicated that the p38 and PI3K/Akt signaling pathways are involved in carbachol-induced ASM cell migration via the M3 mAChR in the A549 cells.

## Discussion

This study demonstrates that the endogenously released acetylcholine stimulates the M3 mAChR in the airway epithelial cells via an autocrine loop and thus leads to the release of IL-8 and TGF-β1, initiating ASM cell migration. Moreover, the p38 and PI3K/Akt signaling pathways are involved in carbachol-induced ASM cell migration via the M3 mAChR in the A549 cells. These findings indicate that the M3 mAChR may be important therapeutic target as it regulates the effects of the epithelial-derived chemokines on ASM cell migration during lung remodeling.

Airway remodeling is the result of tissue injury and repair, and involves the interactions between a variety of structural cells, including epithelial cells and ASM cells. Some growth factors and cytokines have been reported as the chemokines for ASM cells [12, 13, 32]. Cigarette smoking causes direct lung damage, as well as the activation of the lung inflammatory responses and airway remodeling pathways that are important in the etiology of the development of asthma and COPD. The epithelium is a significant source of chemokines, such as cytokines and growth factors. The exposure of epithelial cells to the CSE induced the production of these chemokines, which play an important role in inducing ASM cell migration toward the epithelial surface and contribute to airway remodeling. TGF-β1 and IL-8, important chemokines, are known to be released by epithelial cells following cigarette smoke exposure [33–35]. In the present study, we showed that the CSE-stimulated A549 epithelial cells released IL-8 and TGF-β1, resulting in ASM cell migration toward the epithelial cells. The migratory effect was substantially inhibited by Ac-RRWWCR-NH2 (IL-8 inhibitor) and SB431542 (TGF-β1 inhibitor), suggesting that these chemokines may initiate ASM cell migration.

Interestingly, we have shown that the migration of the ASM cells is induced by the chemokines from the stimulated A549 epithelial cells and could be reduced by treatment with non-selective (tiotropium) and selective (4-DAMP) antagonists of the M3 mAChR. To date, five mAChR subtypes have been identified (M1-M5) and are thought to mediate the majority of the actions of acetylcholine in the peripheral and central nervous systems. Of these, M1, M2 and M3 are expressed at the highest levels in the airways and subserve different physiological functions [36]. The presence of endogenously released acetylcholine stimulates the M3 mAChR via an autocrine loop and thus modulates the functions of the airway epithelial cells, as was previously reported by our and other laboratories [31, 37, 38]. Our observation of the predominant expression of the M3 mRNA by the A549 epithelial cells is consistent with other reports showing that a human bronchial epithelial cell line (16-HBE) expressed both the proteins and mRNAs for the M1, M2 and M3 mAChRs, with the following levels: M3 > M1 > M2 mAChRs [39]. The CSE increases the activity of the chemokines from the human epithelial cells and promotes the cellular response to acetylcholine by affecting the M3 mAChR. The fact that the carbachol-induced migration of the ASM cells toward the A549 epithelial cells blocked by 4-DAMP confirmed the involvement of the M3 mAChR. A synergistic effect of carbachol and CSE on ASM cell migration was also observed in this study. Furthermore, tiotropium could prevent the carbachol (acetylcholine analogue)- and CSE-induced ASM cell migration toward the epithelial surface. This finding further demonstrates that the repertoire of mAChR subtypes in the A549 epithelial cells is modified by CSE stimulation, and the M3 mAChR is significantly up-regulated. Therefore, it is reasonable to assume that the CSE-induced up-regulation of M3 mAChR expression observed in the present study increases the A549 cells’ response to acetylcholine.

The application of the mAChRs antagonists tiotropium or 4-DAMP reduced the CSE-induced ASM cell migration in the absence of exogenous mAChRs ligands or cholinergic neurons in the culture preparations. This finding suggests that the levels of endogenous acetylcholine that are released by the CSE-stimulated A549 epithelial cells are sufficient to exert its autocrine effects via the stimulation of the M3 mAChR. The release of endogenous acetylcholine was also demonstrated by ASM migration toward the epithelial cells treated with the cholinesterase inhibitor neostigmine, which increased the concentration of acetylcholine in the supernatant, suggesting that cholinesterase activities are involved in acetylcholine degradation and constitute an active, endogenous component of the non-neuronal cholinergic system in the process of cells migration. In the A549 cells, the concentration of acetylcholine that is secreted in close proximity to the membrane M3 mAChR is likely to be much higher than that measured in the supernatant. However, these values are similar to the concentrations of exogenous acetylcholine that are required to induce IL-8 release from A549 cells [31] and leukotriene B4 release from 16-HBE cells [38]. In fact, the epithelial cells express the machinery of the cholinergic system, including the acetylcholine-synthesizing choline acetyltransferase, the mAChRs and the acetylcholine-hydrolyzing enzymes acetylcholinesterase and butyrylcholinesterase [31, 37]. Furthermore, the acetylcholine analogue carbachol induced ASM cell migration in a concentration-dependent manner and the carbachol-induced cell migration event could be blocked by tiotropium or 4-DAMP, suggesting that M3 mAChR stimulation is indeed involved in ASM cell migration induced by the chemokines from the stimulated A549 epithelial cells, resulting in airway remodeling. The current scenario strongly suggests a local, autocrine or paracrine role of epithelial acetylcholine in regulating various aspects of airway remodeling via the mAChRs. In addition to synthesizing and releasing acetylcholine, the epithelial cells can also be activated by acetylcholine, which exerts its physiological effects via the activation of M3 mAChR.

The last part of our work focused on the signaling pathways that jointly or individually regulate the ASM cell migration induced by IL-8 and TGF-β1 from the activated A549 epithelial cells in the presence or absence of CSE. IL-8 expression requires the activation of NF-kB and at least one or two MAPK pathways. In addition to the Smad-mediated pathways, other pathways, such as the MAPK and PI3K/Akt pathways, have been implicated in TGF-β1 signaling [40, 41]. Several studies have suggested that the p38, extracellular signal-regulated kinase (ERK) and phosphoinositide 3-kinase (PI3K) signaling pathways play important roles in regulating ASM cell migration [8]. In the present study, we provide evidence regarding the mechanism by which M3 mAChR activation induces IL-8 and TGF-β1 release from the A549 cells through phosphorylation of p38 and Akt, thus causing the ASM cells to migrate toward the epithelial cells. Cell migration was reduced by SB203580 (p38 MAPK inhibitor) and LY294002 (PI3K/Akt inhibitor), respectively, suggesting that p38 and PI3K/Akt signaling pathways were both involved in cell migration. Moreover, the addition of both carbachol and CSE enhanced p38 and Akt phosphorylation and was also inhibited by 4-DAMP, showing that M3 mAChR activation can amplify the signaling pathways that govern the CSE-induced IL-8 and TGF-β1 expression. These findings reinforce and expand other reported experiments showing that the phosphorylation of proteins in the Smad2/3 and ERK pathways is involved in the epithelial-mesenchymal transition triggered by the TGF-β1 from the carbachol-stimulated A549 epithelial cells, resulting in airway remodeling [42]. Moreover, acetylcholine mediates the release of IL-8 in human bronchial epithelial cells by an NF-kB/ERK-dependent mechanism [39], demonstrating the involvement of the p38 and PI3K/Akt signaling pathways in CSE-induced ASM cell migration via the activation of the M3 mAChR.

## Conclusion

In summary, our study provides evidence that the non-neuronal cholinergic system is involved in regulating ASM cell migration. Lung epithelial cells secrete acetylcholine, which may function as an autocrine growth factor via the activation of M3 mAChR, to induce ASM cell migration via the p38 and Akt signaling pathways. These findings demonstrated that the M3 mAChR may be important therapeutic target for obstructive airway diseases, as it regulates the effects of the epithelial-derived chemokines on ASM cell migration, which results in lung remodeling.

## References

[1] Elias JA, Zhu Z, Chupp G, Homer RJ (1999). Airway remodeling in asthma. J Clin Invest.

[2] Hogg JC, Timens W (2009). The pathology of chronic obstructive pulmonary disease. Annu Rev Pathol.

[3] Black JL, Roth M, Lee J, Carlin S, Johnson PR (2001). Mechanisms of airway remodeling. Airway smooth muscle. Am J Respir Crit Care Med.

[4] Stewart AG, Bonacci JV, Quan L (2004). Factors controlling airway smooth muscle proliferation in asthma. Curr Allergy Asthma Rep.

[5] Parameswaran K, Cox G, Radford K, Janssen LJ, Sehmi R, O’Byrne PM (2002). Cysteinyl leukotrienes promote human airway smooth muscle migration. Am J Respir Crit Care Med.

[6] Parameswaran K, Radford K, Fanat A, Stephen J, Bonnans C, Levy BD (2007). Modulation of human airway smooth muscle migration by lipid mediators and Th-2 cytokines. Am J Respir Cell Mol Biol.

[7] Goncharova EA, Billington CK, Irani C, Vorotnikov AV, Tkachuk VA, Penn RB (2003). Cyclic AMP-mobilizing agents and glucocorticoids modulate human smooth muscle cell migration. Am J Respir Cell Mol Biol.

[8] Madison JM (2003). Migration of airway smooth muscle cells. Am J Respir Cell Mol Biol.

[9] Gerthoffer WT (2008). Migration of airway smooth muscle cells. Proc Am Thorac Soc.

[10] Aso H, Ito S, Mori A, Suganuma N, Morioka M, Takahara N (2013). Differential regulation of airway smooth muscle cell migration by E-prostanoid receptor subtypes. Am J Respir Cell Mol Biol.

[11] Takeda N, Sumi Y, Préfontaine D, Al Abri J, Al Heialy N, Al-Ramli W (2009). Epithelium-derived chemokines induce airway smooth muscle cell migration. Clin Exp Allergy.

[12] Chen M, Shi JT, Lv ZQ, Huang LJ, Lin XL, Zhang W (2014). Triptolide inhibits TGF-β1 induced proliferation and migration of rat airway smooth muscle cells by suppressing NF-κB but not ERK1/2. Immunology.

[13] Prakash YS (2013). Airway smooth muscle in airway reactivity and remodeling: what have we learned?. Am J Physiol Lung Cell Mol Physiol.

[14] Foster KA, Oster CG, Mayer MM, Avery ML, Audus KL (1998). Characterization of the A549 cell line as a type II pulmonary epithelial cell model for drug metabolism. Exp Cell Res.

[15] Nardone LL, Andrews SB (1979). Cell line A549 as a model of the type II pneumocyte. Phospholipid biosynthesis from native and organometallic precursors. Biochim Biophys Acta.

[16] Govindaraju V, Michoud MC, Al-Chalabi M, Ferraro P, Powell WS, Martin JG (2006). Interleukin-8: novel roles in human airway smooth muscle cell contraction and migration. Am J Physiol Cell Physiol.

[17] Pera T, Gosens R, Lesterhuis AH, Sami R, Toorn M, Zaagsma J (2010). Cigarette smoke and lipopolysaccharide induce a proliferative airway smooth muscle phenotype. Respir Res.

[18] Knight DA, Holgate ST (2003). The airway epithelium: structural and functional properties in health and disease. Respirology.

[19] Moir LM, Burgess JK, Black JL (2008). Transforming growth factor beta 1 increases fibronectin deposition through integrin receptor alpha 5 beta 1 on human airway smooth muscle. J Allergy Clin Immunol.

[20] Vignola AM, Chanez P, Chiappara G, Merendino A, Pace E, Rizzo A (1997). Transforming growth factor-beta expression in mucosal biopsies in asthma and chronic bronchitis. Am J Respir Crit Care Med.

[21] de Boer WI, van Schadewijk A, Sont JK, Sharma HS, Stolk J, Hiemstra PS (1998). Transforming growth factor beta1 and recruit-ment of macrophages and mast cells in airways in chronic obstructive pulmonary disease. Am J Respir Crit Care Med.

[22] Takizawa H, Tanaka M, Takami K, Ohtoshi T, Ito K, Satoh M (2001). Increased expression of transforming growth factor-beta1 in small airway epithelium from tobacco smokers and patients with chronic obstructive pulmonary disease (COPD). Am J Respir Crit Care Med.

[23] Zwahlen R, Walz A, Rot A (1993). In vitro and in vivo activity and pathophysiology of human interleukin-8 and related peptides. Int Rev Exp Pathol.

[24] Dekkers BG, Maarsingh H, Meurs H, Gosens R (2009). Airway structural components drive airway smooth muscle remodeling in asthma. Proc Am Thorac Soc.

[25] Wessler I, Kilbinger H, Bittinger F, Unger R, Kirkpatrick CJ (2003). The non-neuronal cholinergic system in humans: expression, function and pathophysiology. Life Sci.

[26] Gosens R, Zaagsma J, Meurs H, Halayko AJ (2006). Muscarinic receptor signaling in the pathophysiology of asthma and COPD. Respir Res.

[27] Racké K, Matthiesen S (2004). The airway cholinergic system: physiology and pharmacology. Pulm Pharmacol Ther.

[28] Belmonte KE (2005). Cholinergic pathways in the lungs and anticholinergic therapy for chronic obstructive pulmonary disease. Proc Am Thorac Soc.

[29] Profita M, Giorgi RD, Sala A, Bonanno A, Riccobono L, Mirabella F (2005). Muscarinic receptors, leukotriene B4 production and neutrophilic inflammation in COPD patients. Allergy.

[30] Xu GN, Yang K, Xu ZP, Zhu L, Hou LN, Qi H (2012). Protective effects of anisodamine on cigarette smoke extract-induced airway smooth muscle cell proliferation and tracheal contractility. Toxicol Appl Pharmacol.

[31] Xu ZP, Devillier P, Xu GN, Zhu L, Zhou W, Hou LN (2013). TNF-α-induced CXCL8 production by A549 cells: Involvement of the non-neuronal cholinergic system. Pharmacol Res.

[32] Doherty T, Broide D (2007). Cytokines and growth factors in airway remodeling in asthma. Curr Opin Immunol.

[33] Mio T, Romberger DJ, Thompson AB, Robbins RA, Heires A, Rennard SI (1997). Cigarette smoke induces interleukin-8 release from human bronchial epithelial cells. Am J Respir Crit Care Med.

[34] Halwani R, Al-Muhsen S, Al-Jahdali H, Hamid Q (2011). Role of transforming growth factor-β in airway remodeling in asthma. Am J Respir Cell Mol Biol.

[35] Mortaz E, Henricks PA, Kraneveld AD, Givi ME, Garssen J, Folkerts G (2011). Cigarette smoke induces the release of CXCL-8 from human bronchial epithelial cells via TLRs and induction of the inflammasome. Biochim Biophys Acta.

[36] Barnes PJ (1993). Muscarinic receptor subtypes in airways. Life Sci.

[37] Kummer W, Lips KS, Pfeil U (2008). The epithelial cholinergic system of the airways. Histochem Cell Biol.

[38] Profita M, Bonanno A, Montalbano AM, Ferraro M, Siena L, Bruno A (2011). Cigarette smoke extract activates human bronchial epithelial cells affecting non-neuronal cholinergic system signaling in vitro. Life Sci.

[39] Profita M, Bonanno A, Siena L, Ferraro M, Montalbano AM, Pompeo F (2008). Acetylcholine mediates the release of IL-8 in human bronchial epithelial cells by a NFkB/ERK-dependent mechanism. Eur J Pharmacol.

[40] Hedges JC, Dechert MA, Yamboliev IA, Martin JL, Hickey E, Weber LA (1999). A role for p38(MAPK)/HSP27 pathway in smooth muscle cell migration. J Biol Chem.

[41] Irani C, Goncharova EA, Hunter DS, Walker CL, Panettieri RA, Krymskaya VP (2002). Phosphatidylinositol 3-kinase but not tuberin is required for PDGF-induced cell migration. Am J Physiol Lung Cell Mol Physiol.

[42] Yang K, Song Y, Tang YB, Xu ZP, Zhou W, Hou LN (2014). mAChRs activation induces epithelial- mesenchymal transition on lung epithelial cells. BMC Pulm Med.

